# 

**Published:** 1878-11

**Authors:** 


### CONTENTS.

### COMMUNICATIONS.

"Why do our Fillings Fail?"—*By Dr. I. D. Pearce*........................ 439
Annual Address.—*By Dr. L. G. Noel*........................................ 453
Nævus, or the Effects of Dental Operations Upon the Female During the Period of Utero-Gestation.—*By Dr. E. S. Chisholm*............ 456

### PROCEEDINGS OF SOCIETIES.

A Synopsis of the Proceedings of the American Dental Association, 463

### EDITORIAL.

The Dental Law in Ohio........................................ 476
Ohio State Dental Society........................................ 480
Improvements........................................ 481
Teeth of Gold........................................ 482

